# All-optical fiber optic coherent amplifier

**DOI:** 10.1038/s41598-018-33426-7

**Published:** 2018-10-18

**Authors:** A. Goodarzi, M. Ghanaatshoar, M. Mozafari

**Affiliations:** 1 Laser and Plasma Research Institute, Shahid Beheshti University, G.C., Evin, 1983969411 Tehran, Iran; 2 Advanced Instrumentation Technology Development Center, Niroo Research Institute, Dadman Blvd., 1468613113 Tehran, Iran

## Abstract

A fiber optic-based all-optical amplifier is designed by using the coherent perfect absorption phenomenon. For this purpose, we use a deposited chromium thin layer as an absorbent material on the cross-section of a PM fiber. By placing another fiber in front of the deposited one, we show that by controlling the relative phase between the two counter-propagating beams, total absorbance can be controlled. In the interaction of two beams with unequal intensities, absorption control can be associated with amplification for the weaker beam. By using this mechanism, the effect of an external phase-shifting parameter can be amplified. Furthermore, it is possible to amplify a small signal riding on a CW background through this all-optical procedure.

## Introduction

In data processing and transmission network, data readout encounters major problems created by external noises^1,2^. Particularly, separation of signals from background noises caused by external physical agents such as vibrations is not easy in the optical communications^3,4^. Regularly electronic amplifiers are used to increase the amplitude of an optical signal, however, they not only slow down the process of transferring and information processing, but also they, themselves, receive noises from the surrounding environment (especially, electromagnetic noises)^5–7^. Therefore, develop of all-optical amplifier is necessary to intensify the signal without speed reduction and addition of electromagnetic noises. An all-optical amplifier is a component in which an optical signal is amplified without transforming to an electronic signal. When a small optical signal is entered into this component, a larger-intensity output is created. This component acts as a light intensity controller and is equivalent to an all-optical transistor^8–11^.

For many years, extensive researches have been conducted on the development of all-optical transistors and various samples have been presented based on a variety of mechanisms. Nevertheless, because of the challenges of this path and the failure to meet all the necessary conditions for an operational all-optical transistor^12–14^, research is still underway to design such a piece with acceptable performance. One of these problems is the impossibility of using the designed samples in the form of an integrated optical circuit because of its bulkiness or huge accessories that are necessary for its operation^15^. Insufficient gain is another challenge in design and manufacture of an all-optical transistor. Actually, most of them are just an all-optical switch^16–18^. Cascadability is another important characteristic that is not satisfied by many designed transistors because of using more than one wavelength and the difference between input and output wavelengths^19–21^. On the other hand, the necessity of using high intensity is a fundamental weakness for methods based on nonlinear effects^22–26^.

Among several methods to design all-optical transistors, a group of transistors operates on the basis of coherent properties of light, relative phase and interference^10,11,27–30^. In this type of transistors, the signal to be amplified (gate), along with the pump beam, which provides the required energy for the amplification process, is supplied from a single coherent optical source^31,32^. Despite the fact that the pump and the gate beams are not independent, coherent transistors have wide applications in quantum processing, inline coherent amplifiers and etc.^32–38^.

In this paper, we propose a fiber optic coherent all-optical amplifier that its operation is upon coherent perfect absorption (CPA)^39–41^. The CPA method can be used in several configurations in fiber optics^42,43^. Conventional optical amplifiers are often based on optically active environments^44,45^. In fact, an optically active medium is used as an amplifier, but due to the dependence of the output on the environment condition and the effect of the repetition rate on the active medium population inversion, they cannot be used at very high frequency^46^. Our proposed amplifier works on the basis of counter-propagating waves’ relative phase. We use PM fiber as a light transmission medium and chromium (Cr) thin layer as an absorbent material. We also employ the transfer matrix method (TMM) to calculate the values of primary parameters. In the following, we introduce the designed structure and compare the theoretical and experimental results for the constructed device.

## Results and Discussion

We manage the portion of pump beam which is absorbed within an absorbent material by using the CPA and interference with the gate signal. In this way, it is possible to alter the amplitude of the electric field at the absorbent material place from near zero to a maximum by changing the relative phase of the two counter-propagating optical waves from constructive to destructive interference. Since the amplitude of the absorbed light depends on the light’s electric field vector, the relative phase is a proper parameter to regulate the intensity of the absorbed light. Thus, the light transmission can be controlled by another light wave through this technique.

Most metals such as gold, copper and silver, are good reflectors and absorb a very small part of incident light because of their relatively small real part refractive index. In order to achieve a large absorption, a material with a high absorption coefficient is desired. It should be noticed that in multi-layers or complex structures, like photonic crystals or two dimensional meta-material structures, full absorption could be achieved by a weakly absorbing material. However, in present case, we have not any designed structure. There is just an ultra-thin single layer to restrict the light interaction to a small space to have a simple and integrable system. Such a system requires a high absorbance ultra-thin layer. We chose chromium, which has a big real part of the refractive index, *n*, along with its large imaginary part, *k*. According to the reflection coefficient of a bulk material in normal incidence^47^,

1 $$R=\frac{{({n}_{a}-{n}_{d})}^{2}+{{k}_{a}}^{2}}{{({n}_{a}+{n}_{d})}^{2}+{{k}_{a}}^{2}},$$

where the subscripts “*a*” and “*d*” are for absorber and dielectric media, respectively, Cr will show a large value of absorption and a reduced reflection^11^.

**Figure 1:**
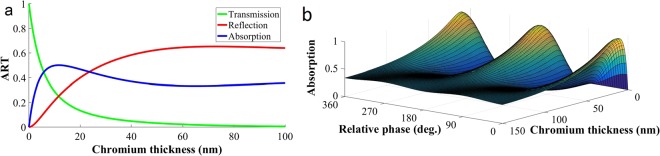
(**a**) Absorption, reflection and transmission values for the Cr thin film surrounded by media with refractive index of 1.45. (**b**) Total absorption of two equal-intensity counter-propagating waves in terms of the relative phase at the position of the Cr layer and the Cr thickness. In small thickness and constructive interference, we have total absorption.

The Cr refractive index in the telecommunication wavelength of 1310 nm is *n* = 4.2 + 4.27*i*^48^. We will place a Cr layer between two optical fibers with the core refractive index of 1.45. Indeed, the Cr layer is deposited on the cross-section of one of them. In this case, the absorption, reflection, and transmission (ART) coefficients for the thin Cr layer can be determined by using the TMM^11,49^. In Fig. 1a, the ART coefficients are plotted for a Cr layer placed between two optical fibers in terms of the layer thickness for a single beam passing through the fibers. It is obvious that the absorbance reaches its maximum value in a thickness of about 17 nm. Getting a large value of absorption will facilitate the intensity variation. In order to determine the influence of the relative phase at the Cr layer position on the absorption, the total absorption is plotted in terms of the relative phase and Cr thickness in Fig. 1b. As can be seen, for the thickness of about 17 nm, full absorption becomes possible for destructive interference at the position of the Cr layer and when the interference is constructive it can reach zero. For large thicknesses of Cr, because of limited penetration length of light, interaction of beams is weak and so the change in absorption is not significant.

Since the interference of two beams occurs through the interaction of their electromagnetic fields and considering the square relation between the electric field amplitude and intensity of a beam, intensity variation can be accompanied by amplification. This concept was firstly pronounced by Zheludev *et al.* in 2015^6^. For a gate beam with the intensity of *I*_*g*_ and the pump beam with the intensity of *I*_*p*_, the following relation governs the interaction of two beams in the adsorbent layer^11^:

2 $${I}_{tot}={({\hat{E}}_{g}\mp {\hat{E}}_{p})}^{2}={\hat{E}}_{p}^{2}+{\hat{E}}_{g}^{2}\mp 2{E}_{p}{E}_{g}cos\theta ={I}_{p}+{I}_{g}\mp 2\sqrt{{I}_{g}{I}_{p}}cos\theta ,$$

where *θ* is the phase difference between electric fields vectors in the absorber layer position. Upon this equation we conclude that it is possible to control the absorption of the pump wave by a low-intensity gate beam. To rate the size of amplification, we define the gain as:

3 $${\rm{G}}{\rm{a}}{\rm{i}}{\rm{n}}=\,\frac{{I}_{tot}-{I}_{p}}{{I}_{g}}=1\mp 2\sqrt{\frac{{I}_{p}}{{I}_{g}}}cos\theta \,.$$

This is similar to the definition of the gain for an electronic transistor.

**Figure 2:**
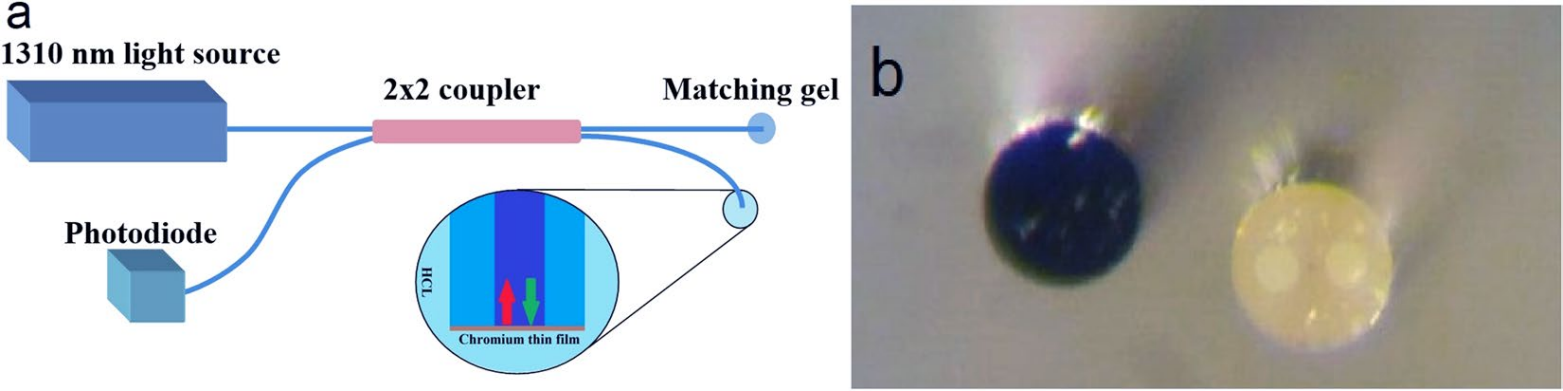
(**a**) The setup for adjusting the thickness of Cr layer and (**b**) PM fiber cross section with and without Cr layer.

To have a well-defined phase at the Cr layer and to achieve complete modulation it is necessary for the wavefront to be parallel with the thin layer and also it is essential that the dimensions of the wavefront be effectively limited^50^. Hence, as previously mentioned, we have used optical fiber as a beam transmitter, as well as a medium for limiting the wavefront. To fulfill our goal, we chose a 125 µm single mode polarization maintaining fiber with a core of 10 µm in diameter. The Cr thin layer was deposited on the cross-section of fiber by using physical vapor deposition method. Then the Cr thickness was adjusted by using the setup illustrated in Fig. 2 and experimental method section.

To investigate the influence of the gate on the pump beam by controlling the coherent absorption, it is necessary to manage the phase of one of the beams through an active manner so that we can adjust the absorption on its maximum or minimum value. This can be done in various ways, such as electro-optical and opto-mechanical phase modulation. We use a cylindrical piezoelectric which resonates with the applied oscillating voltage. The applied harmonic signal will change the optical path by altering the optical fiber length twisted around the piezoelectric cylinder. The variation in optical path depends on the number of turns, frequency, and amplitude of the applied voltage. By this technique, we can modify the relative phase.

**Figure 3:**
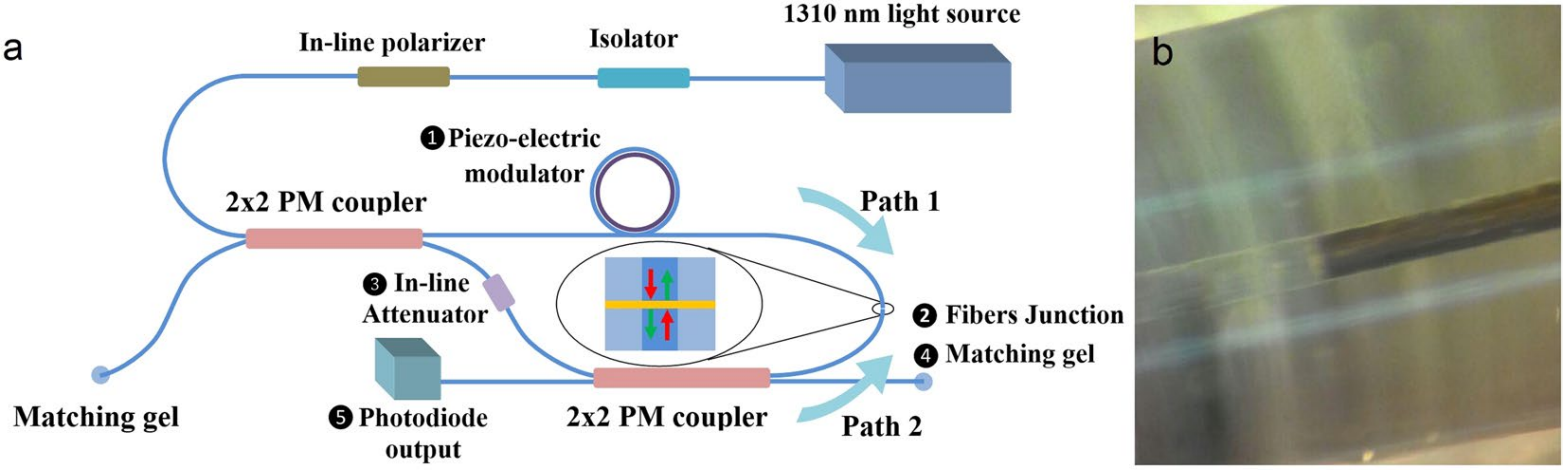
(**a**) The experimental setup for the fiber-based all-optical amplifier. (**b**) Alignment of the coated and uncoated fibers by glass ferrule.

Figure 3a illustrates the setup prepared to investigate the CPA phenomenon and functionality of the proposed technique and Fig. 3b shows fixing method for two fibers. In this setup, a coherent beam passes through an inline fiber optic polarizer and then is divided into two parts by a 50–50 coupler. The beam in the first arm of the first coupler (path 1), which we consider it as the pump beam, after passing through the fiber wrapped around the piezoelectric phase modulator (1), is directed to the bond of the two fibers (2). By using a silica ferrule with 126 µm inner radius, two fibers are placed in front of each other and so, the Cr layer is surrounded between the two fibers. We also eliminate undesirable reflections by using matching gel. The angular direction of the PM fibers is determined by examining the diffraction of a 633 nm laser beam from the cross-section of two fibers. The other part of the input beam from the second arm of the first coupler passes through an inline attenuator (3), and then is divided by a second coupler. The transmitted power through the attenuator can be measured by a power meter in the second arm of the second coupler. The gate is guided from the first arm of the second coupler (4) to the fibers junction and counter-propagates with respect to the pump beam. The interference of these two beams changes the absorption rate of the Cr layer which is measurable at the output (5).

The proposed amplifier is based on coherent light, thus, it is necessary that the two beams be coherent at the Cr layer place. Therefore, the optical paths 1 and 2 must have almost equal length. Actually, the difference in their length must be less than the light source coherence length. The coherence length can be calculated from

4 $$L=2\,\mathrm{ln}(2){\lambda }^{2}/\pi n{\rm{\Delta }}\lambda $$

where λ is the central wavelength, Δλ is the spectral width and *n* is the refractive index of the propagating media^50^. The employed light source is a diode laser with 1310 nm central wavelength and a spectral width of 0.3 nm. Then, from Eq. 4, the coherence length in the fiber is about 1.74 mm. This is absolutely enough to easily control the length difference between the two paths. The reason for the use of two couplers in the setup is to ensure that the signals recorded at the output is only due to the CPA phenomenon in the thin Cr layer. This is guaranteed by the small coherence length in comparison to the arm length.

**Figure 4:**
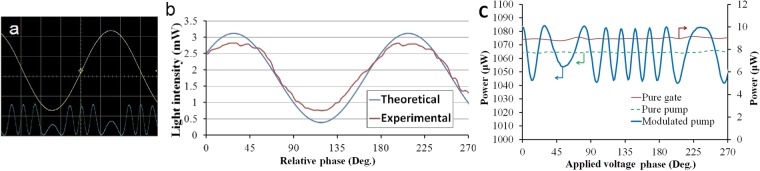
(**a**) Changes in the output intensity for a period of piezoelectric oscillation. (**b**) The modulated output intensity from the experiment and theory versus relative phase at the position of Cr layer. (**c**) The pump modulation amplified by the gate for the pump to gate ratio of 120.

To measure the impact of the gate on the pump beam, we fixed the oscillation frequency at the resonance frequency of the piezoelectric cylinder (about 42 KHz) to achieve maximum oscillation amplitude and to have optical pass variation equivalent to several wavelengths in each cycle. It is expected that for each period of the voltage source, several oscillations in the output intensity are observed. This is a function of the voltage amplitude that is applied to the piezoelectric cylinder. Because this arrangement forms a closed loop, each source of the change in phase produces strong oscillations through interference similar to those of a fiber optic gyroscope. Hence, multi-wavelength optical path variation will stabilize the signal and eliminate the adverse effects of the temperature. Figure 4a shows the intensity of the output beam. For normalization, we consider the intensity of the 2 mW pump as unity. As can be seen, for a period of the applied signal, there are several periods of phase change that is apparent in the output as intensity modulation because of the CPA. In Fig. 4b, the maximum intensity of modulation amplitude has been compared with corresponding theoretical result. It indicates that the Cr thin layer absorption only slightly differs from the obtained theoretical value and as a consequence, the Cr layer actually has a 100% modulation capability through the CPA. The minor incompatibility between theoretical and experimental results is related to the gate and the pump polarization misalignment, the small inaccuracy in the Cr thickness and incomplete coherency. In Fig. 4c the modulated pump beam output is plotted to define amplification characteristic. It is plotted for the pump to gate ratio of 120. For comparison, the pure pump and gate intensities are also depicted in this figure. As can be seen, the modulation amplitude is larger than the gate total intensity.

**Figure 5:**
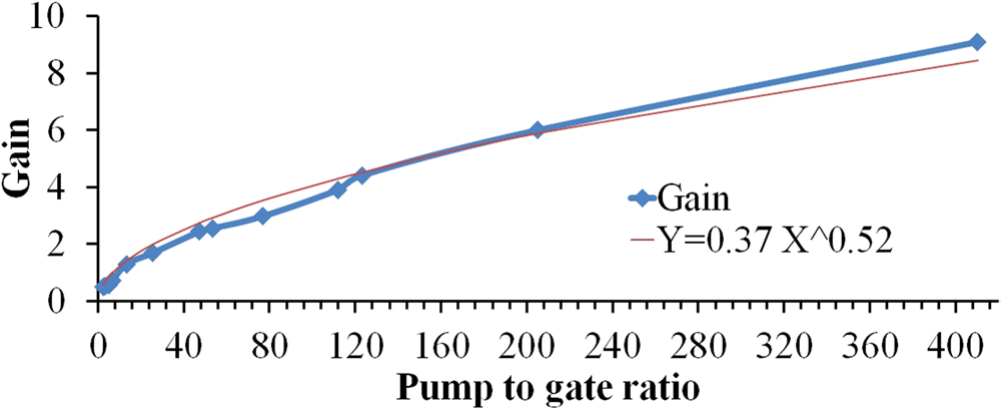
The gain in terms of the pump to gate ratio, accompanied with the fitting curve.

To investigate the effect of the relative intensity of the beams on the gain, the peak-to-peak intensity in output in terms of the pump to gate ratio is depicted in Fig. 5. According to Eq. 3, it is expected that this quantity will not be linear and we will get considerable amplification for small intensities of the gate relative to the pump beam. As can be seen, in the ratio of 400 for the pump to the gate intensity, the peak-to-peak modulation amplitude in the output is about 9.1 times of the gate intensity, i.e. the gain is around 9. It means that the gate beam is able to create a modulated output beam by amplitude higher than itself. It should be noted that the real gain is twice of this amount because the returned beam from the thin layer to the second coupler is divided into two parts and only half of it enters the detector. This amount of gain is approximately one quarter of the theoretical gain calculated from Eq. 3 for this value of pump-to-gate intensity ratio. The difference can arise from unwanted dissipations such as error in controlling Cr layer thickness, coupler deviation from the ideal 50–50 case, semi-coherency as well as the dispersion and reflection from surfaces. In spite of all these losses, the gain that is obtained experimentally is enough to supply input of at least two other transistors.

**Figure 6:**
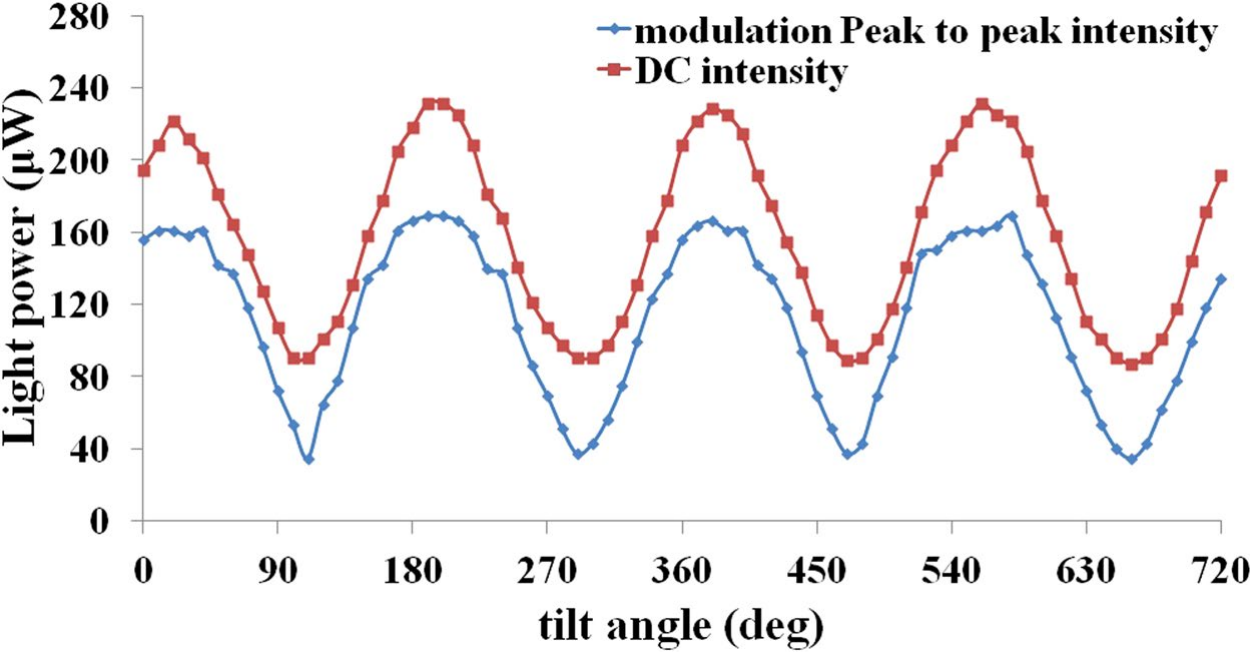
The output DC intensity variation curve and the peak-to-peak amplitude of the output modulation in terms of the off-axis angle of the fibers.

The operation of this transistor is also influenced by the relative polarization direction of the two counter-propagating beams. To study this effect, we plot the amplitude of the output beam for different angles between the two PM fibers polar axis. As can be seen from Fig. 6, the modulation amplitude for the fibers with parallel polar axes is maximal and it decreases with the off-axis angle of the fibers. When the fibers’ polar axes are perpendicular, we still have a modulation. This is because of electric field vectors summation, and it is known that the absorbance quantity depends on the size of the resultant electric field vector.

## Conclusions

The proposed optical amplifier magnifies a small signal riding on a DC beam in a fiber-optic network. We showed that the Cr thin film effectively provides nearly complete absorption conditions through the CPA phenomenon. The gain of this fiber-based system is comparable with that of the electronic transistors. Controllability of the gain is an important characteristic of this amplifier which can be performed by controlling the pump to gate intensity ratio. The obtained gain is enough to derive several similar amplifiers and to use them in a cascade structure. In compare to other all-optical transistors, the CPA provides most of the essential features of an all-optical transistor like operating at room temperature, working by using ultra-low power, cascadability, fast response and integrability. The suggested all-optical fiber-based transistor can be exploited in quantum computing systems.

## Methods

### Chromium layer thickness tuning

To achieve a Cr layer with suitable thickness, we tune a thicker layer to our favorite thickness. For this purpose, after depositing a thick Cr layer by PVD technique, we insert the fiber inside the HCl solution. It should be noted that the proper thickness is firstly determined by simulating the reflection coefficient of the thin layer. Then, by monitoring reflected intensity from the layer in the setup shown in Fig. 2, the thickness could be tuned much finer. A diluted acid may be used to obtain a better result.

## References

[1] Baney DM, Gallion P, Tucker RS (2000). Theory and Measurement Techniques for the Noise Figure of Optical Amplifiers. Opt. Fiber Tech..

[2] Xia, X., Liu, Q., Chen, D. & He, Z. Real-time data processing algorithm for phase-demodulation distributed fiber-optic vibration sensor with signal-to-noise ratio over 30 dB. in 2017 16th International Conference on Optical Communications and Networks (ICOCN) (IEEE, 2017).

[3] Dar R, Feder M, Mecozzi A, Shtaif M (2014). Accumulation of nonlinear interference noise in fiber-optic systems. Opt. Express.

[4] Guo, X., Li, X., Liu, N. & Ou, Z. Y. Quantum information tapping using a fiber optical parametric amplifier with noise figure improved by correlated inputs. *Sci*. *Rep*. **6**, 30214 (2016). 10.1038/srep30214 PMC4960621 27458089

[5] Conforti, E., Ribeiro, N. S. & Gallep, C. M. Speed and Noise Limits of Semiconductor Optical Amplifier Space Switches and Wavelength-Reuse Schemes. in Latin America Optics and Photonics Conference (OSA, 2014).

[6] Fang X, MacDonald KF, Zheludev NI (2015). Controlling light with light using coherent metadevices: all-optical transistor, summator and invertor. Light Sci. Appl..

[7] Slavík R (2010). All-optical phase and amplitude regenerator for next-generation telecommunications systems. Nat. Photonics.

[8] Tiarks, D., Baur, S., Schneider, K., Dürr, S. & Rempe, G. Single-photon transistor using a Förster resonance. *Phys*. *Rev*. *Lett*. **113**, 053602 (2014). 10.1103/PhysRevLett.113.053602 25126919

[9] Xavier SC, Carolin BE, Johnson W, Kabilan AP (2016). Compact photonic crystal integrated circuit for all-optical logic operation. IET Optoelectron..

[10] Goodarzi, A. & Ghanaatshoar, M. Coherent all-optical transistor based on frustrated total internal reflection. *Sci*. *Rep*. **8**, 5069 (2018). 10.1038/s41598-018-23367-6 PMC5864731 29567968

[11] Goodarzi A, Ghanaatshoar M (2016). Controlling light by light: photonic crystal-based coherent all-optical transistor. J. Opt. Soc. Am. B.

[12] Yang X, Hu X, Yang H, Gong Q (2016). Ultracompact all-optical logic gates based on nonlinear plasmonic nanocavities. Nanophotonics.

[13] Hu CY (2017). Photonic transistor and router using a single quantum-dot-confined spin in a single-sided optical microcavity. Sci. Rep..

[14] Miller DAB (2010). Are optical transistors the logical next step?. Nat. Photonics.

[15] Ballarini, D. *et al*. All-optical polariton transistor. *Nat*. *Commun*. **4**, 1778 (2013). 10.1038/ncomms2734 23653190

[16] Liu L, Yue J, Li Z (2017). All-Optical Switch Based on a Fiber-Chip-Fiber Opto-Mechanical System With Ultrahigh Extinction Ratio. IEEE Photonics j..

[17] Stegmaier M, Ríos C, Bhaskaran H, Wright CD, Pernice WHP (2016). Nonvolatile All-Optical 1 × 2 Switch for Chipscale Photonic Networks. Adv. Opt. Mater..

[18] Fang X (2014). Ultrafast all-optical switching via coherent modulation of metamaterial absorption. Appl. Phys. Lett..

[19] Dawes AMC (2005). All-Optical Switching in Rubidium Vapor. Science.

[20] Chen W (2013). All-Optical Switch and Transistor Gated by One Stored Photon. Science.

[21] Paris-Mandoki, A. *et al*. Free-space single-photon transistor based on Rydberg interaction. In Quantum Optics (eds. Stuhler, J. & Shields, A. J.) (SPIE, 2016).

[22] Boixel J (2015). Sequential double second-order nonlinear optical switch by an acido-triggered photochromic cyclometallated platinum(ii) complex. Chem. Commun..

[23] Wang, C.-Y. *et al*. All-optical transistor- and diode-action and logic gates based on anisotropic nonlinear responsive liquid crystal. *Sci*. *Rep*. **6**, 30873 (2016). 10.1038/srep30873 PMC4974645 27491391

[24] Poirier, M. *et al*. In P Integrated Coherent Transmitter for 100 Gb/s DP-QPSK transmission. In Optical Fiber Communication Conference (OSA, 2015).

[25] Fang, X., MacDonald, K. F., Plum, E. & Zheludev, N. I. Coherent control of light-matter interactions in polarization standing waves. *Sci*. *Rep*. **6**, 31141 (2016). 10.1038/srep31141 PMC4981885 27514307

[26] Mousavi SA, Plum E, Shi J, Zheludev NI (2014). Coherent control of birefringence and optical activity. Appl. Phys. Lett..

[27] Mousavi, S. A., Plum, E., Shi, J. & Zheludev, N. I. Coherent control of optical polarization effects in metamaterials. *Sci*. *Rep*. **5**, 8977 (2015). 10.1038/srep08977 PMC4354045 25755071

[28] Plum E, MacDonald KF, Fang X, Faccio D, Zheludev NI (2017). Controlling the Optical Response of 2D Matter in Standing Waves. ACS Photonics.

[29] Zhang J, MacDonald KF, Zheludev NI (2012). Controlling light-with-light without nonlinearity. Light Sci. Appl..

[30] Gholipour B, Zhang J, MacDonald KF, Hewak DW, Zheludev NI (2013). An All-Optical, Non-volatile, Bidirectional, Phase-Change Meta-Switch. Adv. Mater..

[31] Papaioannou M, Plum E, Valente J, Rogers ET, Zheludev NI (2016). Two-dimensional control of light with light on metasurfaces. *Light Sci*. Appl..

[32] Nalla V, Valente J, Sun H, Zheludev NI (2017). 11-fs dark pulses generated via coherent absorption in plasmonic metamaterial. Opt. Express.

[33] Roger, T. *et al*. Coherent perfect absorption in deeply subwavelength films in the single-photon regime. *Nat*. *Commun*. **6**, 7031 (2015). 10.1038/ncomms8031 PMC4455071 25991584

[34] Hi J (2014). Coherent control of Snell’s law at metasurfaces. Opt. Express.

[35] Waters RF, Hobson PA, MacDonald KF, Zheludev NI (2015). Optically switchable photonic metasurfaces. Appl. Phys. Lett..

[36] Chong, Y. D., Ge, L., Cao, H. & Stone, A. D. Coherent perfect absorbers: time-reversed lasers. *Phys. Rev. Lett.* **105**, 053901 (2010). 10.1103/PhysRevLett.105.053901 20867918

[37] Longhi, S. PT-symmetric laser absorber. *Phys. Rev. A* **82**, 031801 (2010).

[38] Baranov Denis G., Krasnok Alex, Shegai Timur, Alù Andrea, Chong Yidong (2017). Coherent perfect absorbers: linear control of light with light. Nature Reviews Materials.

[39] Andrekson, P. A. Phase-sensitive amplifiers for long-haul transmission systems. In 2015 IEEE Photonics Conference (IPC) (IEEE, 2015).

[40] Xomalis, A. *et al*. Fibre-optic metadevice for all-optical signal modulation based on coherent absorption. *Nat*. *Commun*. **9, **182 (2018). 10.1038/s41467-017-02434-y PMC5766546 29330360

[41] Imajuku W, Takada A, Yamabayashi Y (2000). Inline coherent optical amplifier with noise figure lower than 3 dB quantum limit. Electron. Lett..

[42] Rothenberg Jacob M., Chen Christine P., Ackert Jason J., Dadap Jerry I., Knights Andrew P., Bergman Keren, Osgood Richard M., Grote Richard R. (2016). Experimental demonstration of coherent perfect absorption in a silicon photonic racetrack resonator. Optics Letters.

[43] Malara, P. *et al*. Super-resonant intracavity coherent absorption. *Sci. Rep.* **6**, 28947 (2016). 10.1038/srep28947 PMC4929439 27364475

[44] Goel NK, Pickrell G, Stolen R (2014). An optical amplifier having 5cm long silica-clad erbium doped phosphate glass fiber fabricated by “core-suction” technique. Opt. Fiber Tech..

[45] Wang, X. *et al*. High gain submicrometer optical amplifier at near-infrared communication band. *Phys*. *Rev*. *Lett*. **115**, 027403 (2015). 10.1103/PhysRevLett.115.027403 26207503

[46] Wunram M, Storz P, Brida D, Leitenstorfer A (2015). Ultrastable fiber amplifier delivering 145-fs pulses with 6-μJ energy at 10-MHz repetition rate. Opt. Lett..

[47] Hecht, E. Optics, Addison-Wesley (2002).

[48] Rakić AD, Djurišić AB, Elazar JM, Majewski ML (1998). Optical properties of metallic films for vertical-cavity optoelectronic devices. Appl. Opt..

[49] Zamani Mehdi, Ghanaatshoar Majid (2012). Adjustable magneto-optical isolators with flat-top responses. Optics Express.

[50] Bahaa, E. A. S. & Malvin, C. Fundamentals of Photonics, John Wiley & Sons Inc (2009).

